# Comprehensive geriatric assessment in nephrology and the importance of fidelity: what the GOAL trial really tells us

**DOI:** 10.1093/ckj/sfag306

**Published:** 2026-09-02

**Authors:** John A Holland, Judith S L Partridge, Jugdeep K Dhesi

**Affiliations:** School of Immunology and Microbial Sciences, Faculty of Life Sciences and Medicine, King’s College London, London, United Kingdom; Department of Transplant Surgery, Renal medicine and Urology, Guy’s and St Thomas’ NHS Foundation Trust, London, United Kingdom; School of Life Course and Population Sciences, Faculty of Life Sciences and Medicine, King’s College London, London, United Kingdom; Department of Perioperative medicine for Older People undergoing Surgery, Guy’s and St Thomas’ NHS Foundation Trust, London, United Kingdom; School of Life Course and Population Sciences, Faculty of Life Sciences and Medicine, King’s College London, London, United Kingdom; Department of Perioperative medicine for Older People undergoing Surgery, Guy’s and St Thomas’ NHS Foundation Trust, London, United Kingdom

The demographics of those living with kidney disease are changing. Across high-income countries, chronic kidney disease (CKD) is increasingly a condition of later life, with older adults representing the fastest-growing population requiring specialist nephrology care, dialysis, and kidney transplantation [1]. Older patients frequently present to renal services with coexistent frailty, multimorbidity, cognitive impairment, functional dependence, and polypharmacy [2, 3]. These ‘geriatric syndromes’ influence treatment tolerance, decision-making, healthcare utilization, and outcomes, yet they remain incompletely addressed within traditional disease-focused models of nephrology care [4]. Frailty itself remains variably conceptualized and measured in CKD, with different instruments capturing overlapping but distinct constructs and no universally adopted approach to assessment [5].

**Figure 1:**
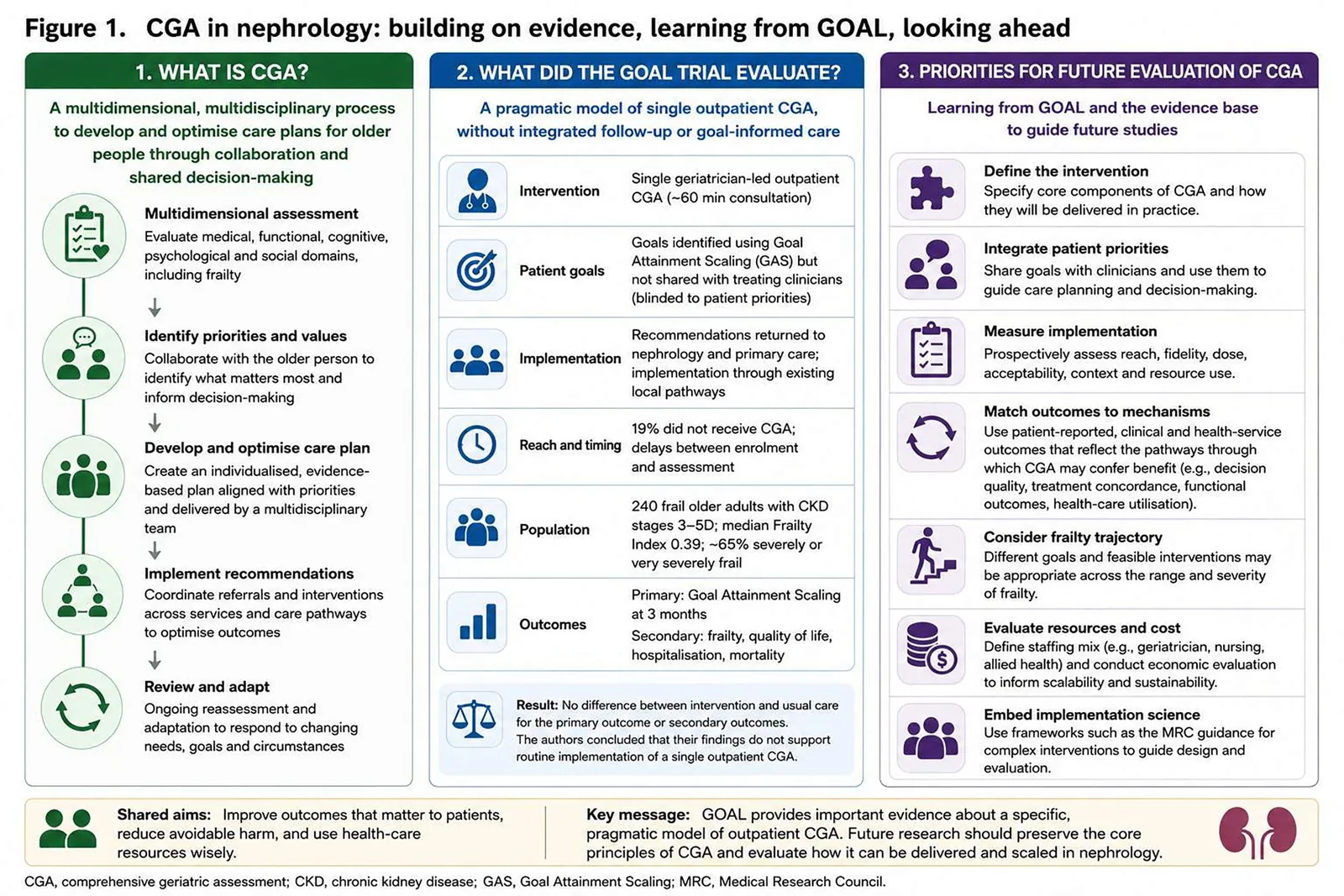
CGA in nephrology: building on evidence, learning from GOAL, looking ahead.

For this reason, comprehensive geriatric assessment and optimization (CGA) has attracted growing interest within nephrology. CGA is a multidimensional, multidisciplinary process through which assessment across medical, functional, cognitive, psychological, and social domains informs individualized care planning, developed collaboratively with older people through shared decision-making (Fig. 1). Decades of evidence from inpatient geriatric medicine, community services, perioperative medicine, and oncology have demonstrated that CGA can improve clinically important outcomes and offer health economic advantage while promoting person-centred care and shared decision-making [6–8]. Although evidence for the role of CGA in those living with CKD is in its infancy, appetite for adapting these principles to nephrology services has grown rapidly.

Against this background, Logan and colleagues should be congratulated for conducting the GOAL trial, the first large pragmatic cluster randomized trial evaluating outpatient CGA in frail, older adults with CKD. Across fifteen Australian renal unit, 240 frail, older adults with CKD stages 3–5D were randomized by unit to receive either a single geriatrician-led outpatient CGA in addition to usual nephrology care or usual care alone. The primary outcome was patient goal attainment at three months, measured using Goal Attainment Scaling (GAS), with secondary outcomes including frailty, quality of life, hospitalization and mortality. The intervention did not improve GAS scores or secondary outcomes, leading the authors to conclude that their findings did not support routine implementation of a single outpatient CGA for frail older adults with CKD [9].

These neutral findings are important in their own right, while also illustrating the inherent complexity of delivering and evaluating multicomponent interventions. The MRC framework for developing and evaluating complex multicomponent interventions emphasizes the need for fidelity to the intervention, embedded stakeholder involvement, outcome measures aligned to the potential impact of the intervention and use of implementation science methodology [10]. The importance of adhering to such a framework has been emphasized in the appraisal of previous CGA studies across clinical settings [11]. The central question raised by the findings of the GOAL study is whether the intervention delivered by the investigators demonstrated fidelity to core CGA components, engaged all stakeholders, used meaningful outcome measures and was underpinned by implementation science principles.

Unlike a pharmacological intervention, CGA is not a discrete treatment but a complex intervention comprising multiple interacting components. Assessment is only the starting point with efficacy dependent on the synthesis of multidimensional information, multidisciplinary collaboration, implementation of coordinated management plans, ongoing review, and adaptation to evolving patient needs. Contemporary frameworks for complex intervention research emphasize that fidelity to these core components is essential if effectiveness is to be meaningfully evaluated [10, 11].

Viewed through this lens, the GOAL intervention intentionally prioritized pragmatism over fidelity. Participants underwent a single outpatient geriatric assessment without planned follow-up, embedded multidisciplinary management, or standardized implementation of recommendations. Subsequent care reflected existing local pathways, with considerable variation in access to allied health services and reliance on primary care clinicians to initiate many recommended interventions. Nineteen percent of participants randomized to the intervention did not receive CGA at all, while a further proportion experienced substantial delays between enrolment and assessment. Although entirely understandable within a pragmatic trial conducted across multiple healthcare settings, these features distinguish the GOAL intervention from the integrated, iterative models in which much of the evidence supporting CGA has been generated. Importantly, they also illustrate the practical challenges of delivering CGA within routine nephrology services: intervention reach, access to multidisciplinary expertise, timeliness, and implementation of recommendations are not simply methodological considerations, but determinants of whether such models can be delivered sustainably in clinical practice. GOAL therefore provides important evidence about both the effectiveness and real-world implementation of this particular model of outpatient CGA.

The comparator also warrants consideration. Experienced nephrology teams caring for an increasingly older and more complex population will already incorporate elements of person-centred care, shared decision-making and geriatric principles within routine practice, potentially reducing the contrast between study groups. This does not diminish the value of CGA, but emphasizes the importance of describing usual care when evaluating the incremental value of complex interventions.

Perhaps the most thought-provoking aspect of GOAL concerns the choice of primary outcome. The use of GAS reflects a commendable commitment to patient-centred care, recognizing that treatment success should be judged according to outcomes that matter to patients rather than clinicians. However, an important methodological tension emerges. To minimize bias, neither the geriatricians nor treating nephrologists were informed of participants' goals, and no structured action planning was undertaken to help patients achieve them. Consequently, clinicians were asked to deliver person-centred care without knowing the outcomes that patients themselves considered most important.

While this decision may be scientifically defensible- and the authors have thoughtfully discussed this issue elsewhere [12]—it illustrates a broader challenge in evaluating complex behavioural interventions. Shared decision-making and goal-directed care are not simply desirable consequences of CGA but fundamental mechanisms through which it is intended to work. By deliberately separating patient goals from clinical management, the trial may have constrained one of CGA’s defining mechanisms: using an individual’s priorities to inform care planning. Measuring goal attainment without integrating those goals into care planning risks evaluating only part of the intervention.

Population selection is equally important. GOAL deliberately recruited a highly frail cohort, with a median Frailty Index of 0.39 and almost two-thirds classified as severely or very severely frail. Frailty measurement in CKD remains heterogeneous, but the deficit-accumulation approach used in GOAL is well established and there is no basis to attribute the neutral findings to instrument selection. More relevant for future studies is whether the timing and objectives of CGA should differ across the frailty trajectory: optimization and functional recovery may be realistic priorities for some patients, whereas shared decision-making, treatment concordance and anticipatory care may assume greater importance with advanced frailty [13].

GOAL highlights broader questions regarding outcome selection for complex interventions in nephrology. Unlike drug trials, where a single biological endpoint may reasonably capture treatment efficacy, complex interventions frequently influence multiple domains simultaneously. Benefits may emerge through improved communication, better coordination of care, greater treatment concordance, enhanced patient confidence, or avoidance of low-value interventions, effects that are not necessarily reflected in a single primary outcome measured over three months. The wider CGA literature has consistently shown that outcomes depend on fidelity to the core intervention, with benefits typically demonstrated in integrated models involving multidisciplinary care planning, longitudinal review, and geriatric expertise [11]. GOAL deliberately evaluated a more pragmatic single-consultation model, and the authors appropriately acknowledge that this may have attenuated effectiveness. Future studies should therefore combine patient-reported, clinical and health-service outcomes with prospective evaluation of implementation, process and context, allowing investigators to understand both intervention effects and how the intervention was delivered.

The GOAL trial should be viewed as an important milestone rather than the final word on CGA in nephrology. It demonstrates the feasibility of conducting large pragmatic trials in frail older adults with CKD while simultaneously emphasizing the challenges of evaluating complex multidisciplinary interventions. Rather than discouraging further investigation, these findings should encourage refinement of intervention design, greater attention to implementation fidelity, and closer integration of nephrology and geriatric medicine principles.

Future studies should be grounded in frameworks such as the MRC guidance for complex interventions and prospectively evaluate fidelity, reach, acceptability, context, and resource requirements alongside clinical outcomes [10]. CGA is inherently multidisciplinary and need not require every component to be delivered by a geriatrician; sustainable models may distribute assessment and management across appropriately trained medical, nursing and allied health professionals while retaining access to geriatric expertise for synthesis and complex decision-making. GOAL itself illustrates the resource implications of this approach, with a geriatrician-led consultation lasting ∼60 min before subsequent implementation through existing services. Economic evaluation should therefore accompany future assessments of scalability. These considerations, together with the core components of CGA and lessons from GOAL relevant to future evaluation, are summarised in Fig. 1.

Integrated models embedding these principles within specific kidney pathways are also being evaluated. Although older kidney transplant candidates constitute a distinct and highly selected population compared with the broader frail CKD population studied in GOAL, the UK-based Older Kidney Patient Optimization Pre-Transplant OK-POP study provides one example, prospectively evaluating feasibility and implementation of CGA within transplant assessment before progression to definitive effectiveness evaluation [14].

Logan and the GOAL investigators have delivered an important study. Their findings remind us that the evaluation of complex interventions extends beyond the intervention itself to the systems, contexts and mechanisms through which it is delivered. As renal services increasingly embrace person-centred care for an ageing population and translate lessons learned about CGA across other specialties and environments, preserving the defining principles of CGA–multidisciplinary collaboration, shared decision-making and coordinated longitudinal care–will be as important as demonstrating effectiveness. The question is not just whether CGA has a role in nephrology, but how it can be delivered and evaluated in ways that preserve its defining principles while remaining feasible within contemporary kidney care.
